# Supplementary material for: The preventative effects of statin on lung cancer development in patients with idiopathic pulmonary fibrosis using the National Health Insurance Service Database in Korea
Source: PLoS One. 2024 Mar 13;19(3):e0299484. doi: 10.1371/journal.pone.0299484 (PMC10936809; doi:10.1371/journal.pone.0299484)
Supplement: S1 Appendix — (DOCX) [file pone.0299484.s001.docx]

**S1 Appendix. Codes list of interstitial lung diseases**

C33 Malignant neoplasm of trachea

C34 Malignant neoplasm of bronchus and lung

D86 Sarcoidosis

M05.1 Rheumatoid lung disease

M05.2  Rheumatoid vasculitis

M05.3 Rheumatoid arthritis with involvement of other organs and systems

M05.8 Other seropositive rheumatoid arthritis

M05.9 Seropositive rheumatoid arthritis, unspecified

M06.0 Seronegative rheumatoid arthritis

M06.8 Other specified rheumatoid arthritis

M06.9 Rheumatoid arthritis, unspecified

M30.1 Polyarteritis with lung involvement [Churg- Strauss]

M31.3 Wegener’s granulomatosis [Necrotizing respiratory granulomatosis]

M31.7 Microscopic polyangiitis

M32  Systemic lupus erythematosus

M33 Dermatopolymyositis

M34 Systemic sclerosis

M35.0 Sicca syndrome [Sjögren]

M35.1 Other overlap syndromes

J84 Other interstitial pulmonary diseases

J84.0 Alveolar and parietoalveolar conditions

J84.1 Other interstitial pulmonary diseases with fibrosis

J84.8 Other specified interstitial pulmonary diseases

J60~J70.9 Lung diseases due to external agents

Statistics Korea, 2023, Nov 4

[http://kssc.kostat.go.kr/ksscNew_web/kssc/common/selectIntroduce.do?gubun=2&bbsId=kcd_ug#](http://kssc.kostat.go.kr/ksscNew_web/kssc/common/selectIntroduce.do?gubun=2&bbsId=kcd_ug)
