# Supplementary material for: The preventative effects of statin on lung cancer development in patients with idiopathic pulmonary fibrosis using the National Health Insurance Service Database in Korea
Source: PLoS One. 2024 Mar 13;19(3):e0299484. doi: 10.1371/journal.pone.0299484 (PMC10936809; doi:10.1371/journal.pone.0299484)
Supplement: Attachment — Submitted filename: Review PLOS 2023.docx [file pone.0299484.s002.docx]

The authors present an epidemiological study with a satisfying sample of patients regarding the impact of statin use in IPF survival. However, there are methodological issues that may have affected research results. Particularly, as the authors do not include lung function parameters and the use of antifibrotics in their registry, important aspects that is known to affect survival in IPF studies limit the significance of their results.

Major comments:

1. You do not include important clinical parameters in IPF patients of both groups, including FVC, DLCO and 6MWT that could have impacted the survival. Lung function would allow us to assess the severity of both groups. I think that this is a serious limitation in the study.
2. The fact that the exercise was significantly different between the statin and the non statin groups could be related to the severity of patients. In case of severe pulmonary fibrosis in which patients need oxygen may have less exercise that other milder cases.
3. The are diverse clinical characteristics that differ between the 2 groups.

Why you do not include all these variables in the multivariate analysis.

4. Did you include age in the multivariate analysis?
5. How could diabetes have affected OS in IPF? Maybe there is a confounding here?
6. Another important limitation is the fact that you have not taken into consideration antifibrotics that affect progression and mortality. So, this may have also affect OS threatening your internal validity.
7. Another limitation, is that you have included patients based on ICD-10, here there might be also misclassification and information bias. It should be also stated in the limitations paragraph.

Minor comments:

1. Please correct in Table 2, 1^st^ row, 3^rd^ column the word ‘statin’.
